# Correlation Between Oxidative Stress and Immune Profiles During Immunotherapy in Metastatic Non-Oncogene-Addicted NSCLC Patients

**DOI:** 10.3390/antiox15030290

**Published:** 2026-02-26

**Authors:** Mariangela Peruzzi, Lucrezia Tuosto, Alain Gelibter, Cristina Nocella, Angela Leonardo, Valentina Magri, Chiara Cataldi, Saula Checquolo, Ilaria Grazia Zizzari, Daniele Santini, Roberto Carnevale, Marianna Nuti, Aurelia Rughetti, Giacomo Frati, Chiara Napoletano

**Affiliations:** 1 Department of Medical and Cardiovascular Sciences, Sapienza University of Rome, 00185 Rome, Italy; mariangela.peruzzi@uniroma1.it (M.P.); cristina.nocella@uniroma1.it (C.N.); 2 Maria Cecilia Hospital, GVM Care & Research, 48010 Cotignola, Italy; 3 Department of Experimental Medicine, Sapienza University of Rome, Regina Elena 324, 00161 Rome, Italy; lucrezia.tuosto@uniroma1.it (L.T.); ilaria.zizzari@uniroma1.it (I.G.Z.); marianna.nuti@uniroma1.it (M.N.); aurelia.rughetti@uniroma1.it (A.R.); 4 Division of Medical Oncology, Department of Radiological, Oncological, and Pathological Sciences, “Sapienza” University of Rome, 00185 Rome, Italy; ang.leonardo.28@gmail.com (A.L.); valentina.magri@uniroma1.it (V.M.); chiara.cataldi@uniroma1.it (C.C.); daniele.santini@uniroma1.it (D.S.); 5 Department of Medical and Surgical Sciences and Biotechnologies, Sapienza University of Rome, 04100 Latina, Italy; saula.checquolo@uniroma1.it (S.C.); roberto.carnevale@uniroma1.it (R.C.); giacomo.frati@uniroma1.it (G.F.); 6 IRCCS Neuromed, 86077 Pozzilli, Italy

**Keywords:** NOX2, sNOX2-dp, oxidative stress, immunotherapy, NSCLC, anti-PD1 antibodies

## Abstract

Oxidative stress is considered one of the cancer hallmarks, influencing tumor initiation, progression, and metastasis. High levels of reactive oxygen species (ROS) impair the effectiveness of the immune response in cancer patients. We examined changes in oxidative stress during immunotherapy, exploring the relationship between the immune system and clinical parameters related to oxidative burden. Several T-cell and myeloid subsets from 79 metastatic non-oncogene-addicted non-small-cell lung cancer (NSCLC) patients were analyzed using flow cytometry. Additionally, 20 cytokines were measured in serum samples, and sNox2-dp levels, an indicator of NOX2 activity, were assessed by ELISA. Seventy-nine healthy donors served as controls. The data showed that cancer patients had higher levels of sNox2-dp compared to healthy donors (*p* < 0.0001). Elevated sNox2-dp levels were associated with inflammation-related comorbidities (*p* = 0.008) and platelet counts (*p* = 0.03) in NSCLC patients. Furthermore, sNox2-dp displayed a negative correlation with immune cells involved in activation, such as proliferating (Ki67^+^) CD8^+^, PD1^+^ and effector lymphocytes, and a positive correlation with immunosuppressive PMN-MDSCs and inflammatory soluble immune factors, including IL1α, IL1β, IL6, IL10, CCL3, and CCL4. Oxidation levels decreased after immunotherapy (*p* = 0.04) and increased only in non-responder patients (*p* = 0.02). Oxidative stress may be indirectly affected by immunotherapy and could serve as a novel tool to identify responding patients in the NSCLC setting.

## 1. Introduction

The Nicotinamide Adenine Dinucleotide Phosphate (NADPH) oxidase (NOX) is a family of enzymes that generate reactive oxygen species (ROS). The NOX family includes seven isoforms (NOX1, NOX2, NOX3, NOX4, NOX5, DUOX1, DUOX2), which share structural similarities but differ in tissue distribution and regulatory mechanisms [1]. Specifically, NOX2 is expressed in lysosomal and myeloid cells, where it aids in killing phagocytosed microbes, supporting pathogen destruction and regulating neutrophilic inflammation to protect the host from damage caused by persistent inflammation [2]. This enzyme is activated by cytokines, oxidative stress, or immune signals and transfers electrons from NADPH to molecular oxygen, producing ROS (H_2_O_2_, O_2_^−^, OH^−^, and ONOO^−^) [2]. Excessive activation of NOX2 links to many diseases, including cancer, due to overproduction of ROS and oxidative damage [3,4]. In the tumor microenvironment, ROS influence the behavior of cancer and stromal cells, affecting tumor progression and survival [4,5]. ROS can induce oncogenic changes by damaging DNA and influence epigenetic regulation of gene expression, thus promoting tumor cell proliferation [4]. Conversely, they can also prevent tumor development by inducing cell senescence or ROS-dependent cell death, which helps eliminate cancer cells. Additionally, ROS play varying roles in controlling metastasis formation. They can promote metastasis by affecting metalloprotease production [6], aiding in *invadopodia* formation [7], and encouraging the epithelial–mesenchymal transition [8]. On the other hand, elevated ROS levels during tumor progression may become harmful to cancer cell survival, causing loss of matrix attachment and leading to cell death [9].

ROS produced inside and outside cells can act as key regulators in the interaction between tumor cells and immune cells within the stroma [10]. Immune cells can either release ROS or be directly affected by these molecules. ROS from neutrophils can cause DNA damage that promotes cancer [11], or it can also prevent tumor metastasis in solid tumors by inducing ferroptosis [12]. Myeloid-Derived Suppressive Cells (MDSCs), including Polymorphonuclear MDSCs (PMN-MDSCs) and Monocyte-MDSCs (M-MDSCs), along with Tumor-Associated Macrophages (TAMs), release ROS as a mechanism of immunosuppression, which limits the anti-tumor immune response [13,14]. Elevated levels of ROS can also impact T-cell activity by inducing an exhausted T-cell phenotype, thereby favoring tumor progression [15,16], enhancing regulatory T-cell (Treg) functions, or promoting T-cell death. Additionally, PD1 signaling triggers apoptosis in alloreactive T cells and increases cellular ROS levels. This increase depends on fatty acid oxidation [17], making PD-1 blockade a potential strategy to inhibit ROS production. These data emphasize the dual role of ROS in tumor development, illustrating the complex relationship between these molecules, cancer, and immune cells.

In this study, we examined the oxidative systemic burden caused by NOX2 activity in 79 patients with metastatic non-oncogene-addicted non-small-cell lung cancer (NSCLC) at baseline and after one cycle of anti-PD1-based immunotherapy. We measured the release of the soluble NOX2-derived peptide (sNOX2-dp) in patients’ sera to reflect NOX2 activity [18]. Simultaneously, we assessed the patients’ immune systems, which were correlated with sNOX2-dp release and clinical parameters.

## 2. Materials and Methods

### 2.1. Patients’ Characteristics and Criteria of Inclusion

Seventy-nine patients with advanced, non-oncogene-addicted non-small-cell lung cancer (NSCLC) were enrolled at Policlinico Umberto I in Rome. Inclusion criteria included: age over 18 years; histologically confirmed diagnosis of NSCLC; Eastern Cooperative Oncology Group (ECOG) performance status of 2 or less; measurable disease; and adequate pulmonary, cardiac, renal, liver, and bone marrow function. Patients with stable, asymptomatic central nervous system metastases were eligible. Exclusion criteria included autoimmune disease; symptomatic interstitial lung disease and other significant comorbidities; systemic immunosuppression; and prior treatment with immune-stimulatory anti-tumor agents, including checkpoint-targeted therapies. Before therapy began, patients were screened for EGFR, ALK, and ROS1 mutations, and all results were negative. After genetic screening, patients received anti-PD1 therapy, which included Pembrolizumab alone when tumor PD-L1 expression was 50% or higher, and Pembrolizumab or Nivolumab/Ipilimumab (anti-PD1 and CTLA-4 antibodies, respectively) combined with chemotherapy when PD-L1 was below 50%. Toxicity was reported according to the Common Terminology Criteria for Adverse Events (version 4.0) and was assessed on day 1 of each cycle through the end of treatment. The disease control rate (DCR) was assessed in each patient and used to classify patients as responders (R) or non-responders (NR). R showed a complete or partial response, or stable disease, according to iRECIST criteria, while NR showed progression as the best response after three months of treatment. The study was conducted in accordance with good clinical practice guidelines and the Declaration of Helsinki, and it received approval from the Ethics Committee of Policlinico Umberto I (Ethical Committee Protocol, RIF.CE: 4181).

### 2.2. Peripheral Blood Mononuclear Cell and Serum Collection

Peripheral blood mononuclear cells (PBMCs) and sera were collected from 79 NSCLC patients who received immune checkpoint inhibitors (ICIs) as first-line therapy at baseline (T0) and after 3 weeks of treatment (T1). PBMCs were isolated from blood samples using Ficoll–Hypaque gradient (Lympholite-H, Burlington, ON, Canada) by centrifugation at 1800 rpm for 30 min. Sera from patients and 79 healthy donors were obtained using BD Vacutainer Plus Plastic Serum tubes (Becton Dickinson, Franklin Lakes, NJ, USA) by centrifugation at 1800 rpm for 10 min. All samples were cryopreserved until use.

### 2.3. Immunophenotype

PBMCs were analyzed using a multi-parametric approach with conjugated anti-human monoclonal antibodies (MoAbs) to identify 52 cellular subsets, as described previously [19]: 1. T-cell subsets with anti-CD3-BV510 (HIT3a clone), CD8-APC-H7 (SK1 clone), Ki67-BV420 (B56 clone), CD137 (4-1BB)-APC (4B4-1 clone), PD1-BB700 (EH12.1 clone) (all from BD Biosciences, San Jose, CA, USA), and CD45RA-BB515 (HI100 clone) (BD Biosciences) combined with CCR7-PE (G043H7 clone) (BioLegend, San Diego, CA, USA) for identifying naïve, central memory, effector, and effector memory T-cell subpopulations; 2. Regulatory T cells with anti-CD3-BV510 (HIT3A clone), anti-CD4-APC-H7 (RPA-T4 clone), CD45RA-BB515 (HI100 clone) (all from BD Biosciences), CD25-PE (MA251 clone) (BioLegend), and FOXP3-APC (PCH101 clone) (Thermo Fisher Scientific, Waltham, MA, USA); and 3. MDSCs (PMN- and M-MDSC) with CD45-AF700 (2D1 clone), HLA-DR-FITC (L243 clone), CD14-BB700 (MφP9 clone), CD15-APC (HI98 clone), and LOX1-PE (15C4 clone) (all from BioLegend), along with CD66b-PECy7 (DREG-5 clone, BD Biosciences). Negative controls were obtained using the Fluorescence Minus One (FMO) strategy. Live cells were identified using the Zombie Aqua Fixable Viability Kit (BioLegend), and intracellular staining was performed with the Foxp3/Transcription Factor Staining Buffer Set (Invitrogen, Waltham, MA, USA). The different Treg populations (CD4^+^CD25^+^) were defined by the expression of FOXP3 and CD45RA markers, as previously described by Myiara et al. [20]. Resting Tregs were identified as CD45RA^+^FoxP3^lo^, activated Tregs as CD45RA^−^FoxP3^hi^, and non-suppressive Tregs as CD45RA^−^FoxP3^lo^. PMN-MDSCs were identified by gating on CD45^+^ cells, then selecting for CD66b^+^HLA-DR^−^ markers. M-MDSCs were recognized by gating on CD45^+^ cells, followed by selecting for CD66b^−^ cells, CD14^+^HLADR^−^, as previously described by Scirocchi et al. [21]. All samples were acquired on the DxFLEX Flow Cytometer (Beckman Coulter, Brea, CA, USA) and analyzed with FlowJo (version 10.8.8, Becton Dickinson).

### 2.4. Soluble Factors

Serum soluble cytokines were measured in cancer patients using the Inflammation 20-Plex Human ProcartaPlex Panel (Thermo Fisher Scientific) according to the manufacturer’s instructions. This panel includes 20 cytokines and immune molecules (sE-Selectin, GM-CSF, ICAM/CD54, IFNα, IFNγ, IL1α, IL1β, IL4, IL6, IL8, IL10, IL12p70, IL13, IL17A/CTLA8, IP10/CXCL10, MCP1/CCL2, MIP1α/CCL3, MIP1β/CCL4, sP-Selectin, TNFα) evaluated through Luminex multiplex assays (ThermoFisher Scientific) and analyzed with the Bioplex Manager MP software (Version 6.2, Bio-Rad, Hercules, CA, USA).

### 2.5. sNOX2-dp Analysis

NOX2 activation was assessed by detecting soluble NOX2-derived peptide (sNOX2-dp) using an ELISA, as described previously [18]. The peptide is identified by a monoclonal antibody that binds to the extramembrane segment of NOX2 (224–268), which is released upon activation. Enzyme activity is then measured spectrophotometrically by monitoring the increase in absorbance at 450 nm after acidification of the reaction products with 2 M sulfuric acid. Values are expressed in pg/mL; intra-assay and inter-assay coefficients of variation were 8.95% and 9.01%, respectively.

### 2.6. Statistical Analysis

Student’s *t*-tests, both paired and unpaired, were used to examine groups of quantitative variables, shown as mean ± standard error of the mean (SEM). A *p*-value under 0.05 was considered statistically significant. Correlations between sNox2-dp and immune cell subsets were assessed through Spearman’s rank correlation test. Data collection and analysis were carried out using GraphPad Prism version 10 (GraphPad Software, Inc., San Diego, CA, USA).

## 3. Results

### 3.1. Patients’ Characteristics

Seventy-nine patients diagnosed with metastatic non-oncogene-addicted NSCLC were enrolled in this study; their characteristics are summarized in Table 1. The median age was 67 years (range 48 to 90 years). Most patients were male (65%), while 35% were female. The performance status (PS), which reflects patients’ clinical condition, showed that 53% of patients with optimal health had PS = 0, whereas 47% with declining health conditions were PS = 1 (39%) and PS = 2 (8%). Thirty-four patients were current smokers, and 36 were former smokers. Non-smokers accounted for 11% of the cohort. Fifty patients had comorbidities. All patients received anti-PD1-based immunotherapy alone (39%) or combined with chemotherapy (61%) as their first-line treatment. Fifty-two patients were classified as responders (R), while 27 were non-responders (NR) after three months of therapy.

### 3.2. High sNOX2-dp Levels Are Associated with Comorbidities and Platelet Counts in NSCLC Patients

**Figure 1:**
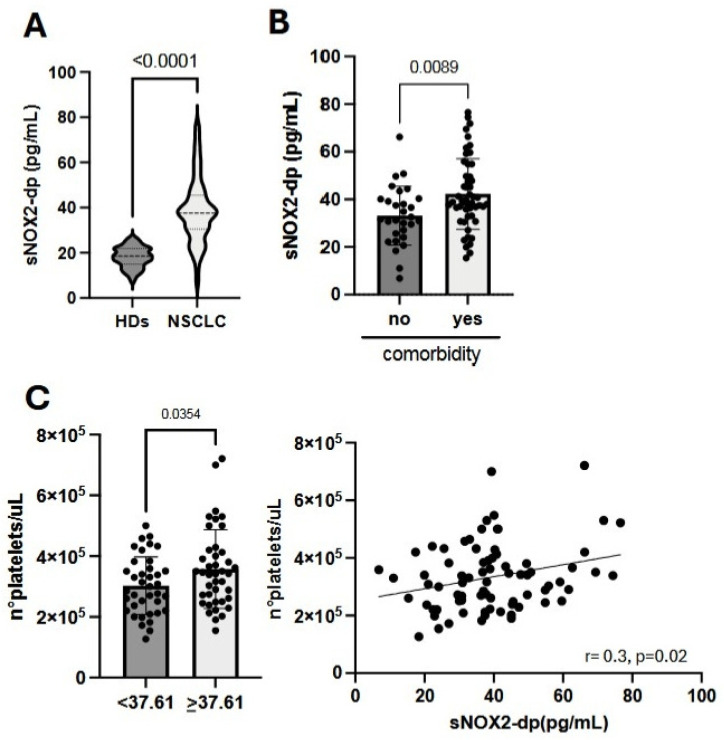
Evaluation of sNOX2-dp levels and their correlation with comorbidities and platelet counts. (**A**) Violin plots show the concentration of sNOX2-dp in 79 healthy donors (HDs) and 79 NSCLC patients (NSCLC) evaluated at baseline. (**B**) Histograms display the levels of sNOX2-dp in patients without (no) and with (yes) inflammation-related comorbidities ± SEM. (**C**) Histograms illustrate the absolute number of platelets based on the median sNOX2-dp level (37.61 pg/mL) ± SEM (**left**), and the correlation between platelet count and sNOX2-dp levels (**right**). *p*-values < 0.05 were considered significant.

To understand the role of sNOX2-dp in NSCLC patients undergoing immunotherapy, we first compared sNOX2-dp levels at baseline in cancer patients (CPs) with those in 79 healthy donors (HDs) (Figure 1A). The results showed that NSCLC patients had significantly higher sNOX2-dp levels than HDs (*p* < 0.0001), indicating elevated oxidation levels in CPs associated with cancer-related inflammation. We further examined the relationship between serum sNOX2-dp concentration and various clinical parameters. We found high levels of sNOX2-dp in patients with previous comorbidities, mainly inflammatory conditions affecting the lung (30%), articulation (8%), circulatory system (4%), and gastrointestinal tract (10%) (*p* = 0.0089), along with elevated platelet counts (*p* = 0.0354) (Figure 1B,C). No correlations were observed between sNOX2-dp and other clinical factors, as summarized in the Supplementary Table S1.

### 3.3. sNOX2-dp Negatively Correlates with Immune System Activation and Is Positively Associated with an Inflammatory Milieu

**Figure 2:**
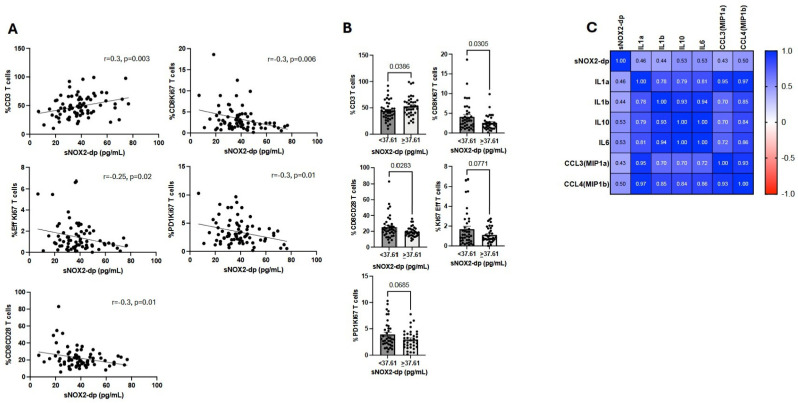
sNOX2dp correlates with immune cells and inflammatory cytokines. (**A**) Correlations between sNOX2dp and CD3^+^, CD8^+^Ki67^+^, Eff Ki67^+^, PD1^+^Ki67^+^ and CD8^+^CD28^+^ T cells. (**B**) Histograms represent the percentage of the median values ± SEM of CD3^+^, CD8^+^Ki67^+^, Eff Ki67^+^, PD1^+^Ki67^+^ and CD8^+^CD28^+^ T cells grouped based on the median value of sNOX2-dp (37.61 pg/mL). (**C**) Heat map representing the correlation between sNOX2-dp and soluble factors. The values in each square correspond to Pearson r values. *p*-values < 0.05 were considered significant.

To better assess the impact of sNOX2-dp on the immune system, we correlated this parameter with various immune cell subsets across the entire NSCLC patient population (Figure 2). Data from this analysis showed that sNOX2-dp was directly linked to the percentage of CD3+ T cells but negatively correlated with several immune cell subsets involved in initiating an anti-tumor immune response. These included proliferating (Ki67^+^) cytotoxic (CD8^+^), effector (CD45RA^+^CCR7^−^), and activated (PD1^+^) T-cell subsets, as well as the cytotoxic T-cell population expressing the CD28 costimulatory molecule (Figure 2A). A similar analysis was performed by dividing NSCLC patients based on treatment groups (PEM, PEM+CHT, and Ipi/Nivo+CHT) (Supplementary Table S2). Results confirmed the findings observed in the overall patient population, involving different cellular subsets but with similar functions. Specifically, CD3+ T cells evaluated at T0 directly correlated with sNOX2-dp levels at baseline in patients treated with PEM and Ipi/Nivo+CHT. Conversely, activated T cells, such as effector memory T cells in the PEM group, various CD137 subsets in the PEM+CHT group, and different proliferating T-cell subsets in the Ipi/Nivo+CHT group, were inversely correlated with sNOX2-dp. Additionally, when we analyzed immune cells of the entire population using the median sNOX2-dp value of 37.61 pg/mL, we found that patients with sNOX2-dp levels at or above 37.61 pg/mL had an increased percentage of CD3^+^ cells and decreased levels of activating T-cell subsets (CD8^+^Ki67^+^, Effector Ki67^+^, PD1^+^Ki67^+^, and CD8^+^CD28^+^ T cells) (Figure 2B). A similar analysis was performed considering the different therapies. sNOX2-dp values below 37.61 were associated with higher levels of CD8+Ki67+ T cells in patients treated with PEM; higher percentages of CD137^+^ and CD137^+^Ki67^+^ T cells in the PEM+CHT group; and lower levels of CD3^+^ cells in patients who underwent Ipi/Nivo+CHT (Supplementary Figure S1). All these analyses support the idea that higher sNOX2-dp levels are linked to suppressed immune responses. Finally, the assessment of circulating cytokines in cancer patients revealed a direct correlation between sNOX2-dp and several cytokines involved in maintaining the tumor inflammatory microenvironment, such as IL1 α (*p* = 0.01) and β (*p* = 0.02), IL6 (*p* = 0.004), as well as the macrophage inflammatory chemokine ligands CCL3 (*p* = 0.02) and CCL4 (*p* = 0.004), and the inhibitory cytokine IL10 (*p* = 0.005) (Figure 2C).

### 3.4. Circulating sNOX2-dp Levels Decrease During Immunotherapy and Correlate with Treatment Response

**Figure 3:**
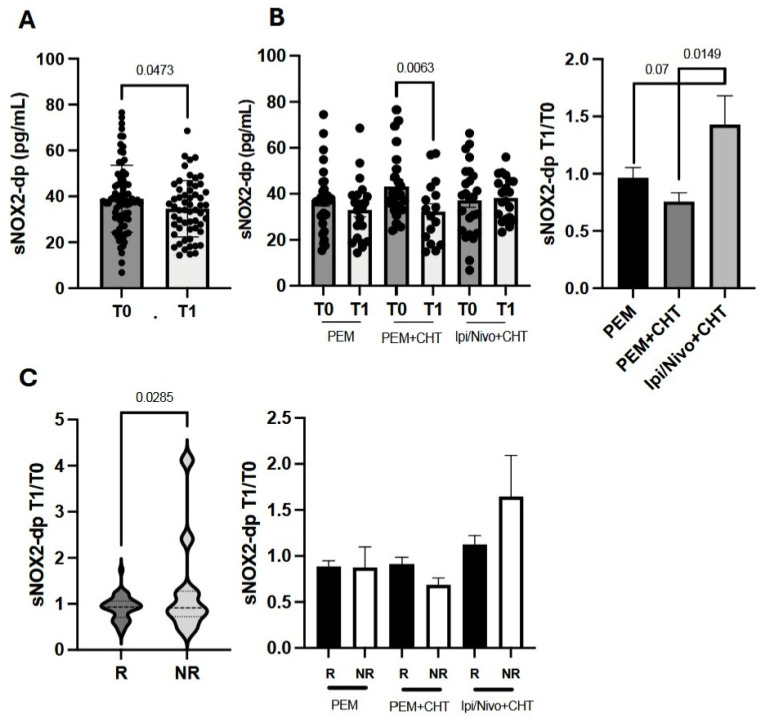
sNOX2-dp changes throughout therapy and its relationship with treatment and response. (**A**) Histograms show the median value of sNOX2-dp ± SEM at baseline (T0) and after one cycle of therapy (T1); (**B**) histograms represent the median value of sNOX2-dp ± SEM at baseline (T0) and after one cycle of therapy (T1) based on therapy (left) and the median values of the pNox1fold increase evaluated as ratio T1/T0 ± SEM (**right**); (**C**) violin plots show the sNOX2-dp fold increase evaluated in responders (R) and non-responders (NR) patients (**left**); histograms represent the median value of pNox1 ratio T0/T1 ± SEM based on treatment. *p*-values < 0.05 were considered significant. PEM: pemigatinib; CHT: chemotherapy; Ipi: ipilimumab; Nivo: Nivolumab.

To assess how sNOX2-dp levels change during immunotherapy, we first measured sNOX2-dp after the initial treatment cycle (T1) across the entire NSCLC patient group. Results showed a small but statistically significant decrease in sNOX2-dp at T1 (Figure 3A). The modulation of sNOX2-dp was also examined among the three treatment options (Figure 3B). While sNOX2-dp levels were similar across all three groups at T0, measurements at T1 revealed a significant reduction only in the group treated with PEM plus CHT (*p* = 0.0063) (Figure 3B, left panel). Additionally, when sNOX2-dp was analyzed as a fold change between T1 and T0, data indicated that Ipi/Nivo+CHT caused a significant increase in sNOX2-dp at T1 compared to PEM+CHT (*p* = 0.0149). A similar trend was observed when comparing Ipi/Nivo+CHT to PEM, although it was not statistically significant (*p* = 0.07). Conversely, sNOX2-dp modulation was comparable in patients treated with PEM and PEM+CHT, suggesting that chemotherapy did not affect sNOX2-dp levels in these groups (Figure 3B, right panel).

Changes in sNOX2-dp were also analyzed based on therapy response and correlated with immune cell levels at T0. This approach helps determine whether baseline immune cell percentages influence sNOX2-dp modulation. Data from the entire NSCLC cohort showed that non-responders (NR) experienced a significantly greater increase in sNOX2-dp at T1 compared to responders (R) (*p* = 0.0285). However, when analyzing the three treatment groups separately, no differences between R and NR were observed (Figure 3C). When the sNOX2-dp fold increase was correlated with baseline immune cell percentages (Supplementary Table S3), we found that the fold increase was mainly inversely related to naïve T cells or activated lymphocyte subsets, further confirming this.

### 3.5. The Increase in sNOX2-dp Fold Change Correlates with Low Levels of Non-Suppressive Tregs and High MDSC Levels

**Figure 4:**
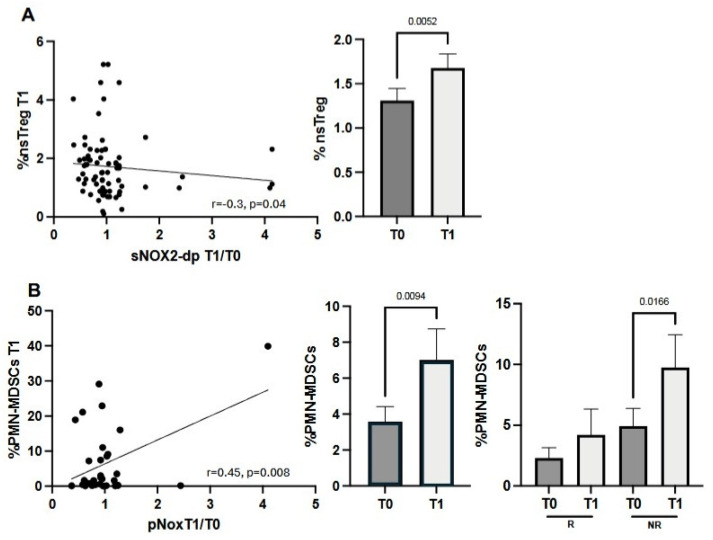
sNOX2-dp correlated with non-suppressive Tregs (nsTreg) and PMN-MDSCs. (**A**) Correlation between the fold increase in sNOX2-dp from T0 to T1 and the percentage of non-suppressive Tregs at T1 (**left**); histograms show the median values of nsTregs from T0 to T1 ± SEM (**right**). (**B**) Correlation between the T1/T0 sNOX2-dp ratio and the percentage of PMN-MDSCs at T1 (**left**); histograms represent the median values of PMN-MDSCs at baseline and after one cycle ± SEM (left histograms) and based on response to therapy (right histograms). *p*-values < 0.05 were considered significant.

We finally examined the relationship between sNOX2-dp levels and immunosuppression, assessed at T0 and T1 by the percentage of Tregs (active, resting, and non-suppressive subsets) and MDSCs, including both PMN- and M-MDSCs (Figure 4). The only Treg subset that negatively correlated with an increase in sNOX2-dp fold change was the non-suppressing Tregs evaluated at T1, which increased during treatment (Figure 4A, right panel). Additionally, we found that PMN-MDSCs evaluated at T1, but not M-MDSCs, positively correlated with rising sNOX2-dp levels (Figure 4B). In fact, when analyzing PMN-MDSC levels, the percentage of these cells increased at T1 (*p* = 0.0094) (Figure 4B, middle panel). This increase was mainly observed in NR patients (Figure 4B, right panel), suggesting that higher sNOX2-dp levels might be associated with immunosuppression.

## 4. Discussion

The oxidative status of cancer patients is crucial in tumor growth, from initiation to progression and metastasis [10]. This condition also affects the effectiveness of the anti-tumor immune response, making oxidative stress a key feature of cancer. Combining therapies that target the immune system and oxidative pathways could be an effective strategy to enhance the anti-tumor immune response and improve outcomes for cancer patients.

In this study, we evaluated the oxidative status of 79 metastatic, non-oncogene-addicted NSCLC patients by measuring sNOX2-dp levels in their sera. sNOX2-dp reflected NOX2 activity involved in ROS release [3]. These assessments were conducted at baseline and after one cycle of anti-PD1-based immunotherapy. Simultaneously, we analyzed several immune cell subsets that regulate the anti-cancer response to understand the relationship between NOX2 activity and immune cells. A control group of 79 healthy donors was included for comparison of sNOX2-dp release. The analysis showed that, at baseline, cancer patients exhibited higher serum sNOX2-dp levels than healthy individuals, indicating increased ROS levels in patients. Indeed, ROS plays a crucial role in the development of chronic inflammation and tumor metastasis, which are characteristic of the advanced stage in these enrolled patients [22]. We also examined the correlation between clinical parameters and baseline sNOX2-dp levels. The absolute number of platelets and the presence of comorbidities were associated with elevated NOX2 activity. Furthermore, platelets are a source of ROS; when activated, they trigger NOX1/2 signaling, which increases ROS generation by NOX and causes a significant redox imbalance that further amplifies ROS production and leads to additional platelet activation [23]. Additionally, most patients with comorbidities had chronic or previous inflammation-related diseases, such as emphysema, diabetes, or heart disease, which contributed to enhanced ROS production [24,25,26].

When we combined the analysis of several immune cell subsets with the sNOX2-dp serum concentration, we found that high levels of sNOX2-dp (>37.61 pg/mL) were associated with a slight increase in CD3^+^ T cells. However, analysis of these lymphocytes showed that they did not belong to activated subsets. In fact, the percentage of cytotoxic and proliferating effector T cells was elevated only in patients with low sNOX2-dp levels (<37.61 pg/mL). Similar results appeared when examining the effects of each treatment on the immune system. Cytotoxic, proliferating, and tumor-specific T cells decreased with high sNOX2-dp levels. Measuring serum soluble immune factors also showed that sNOX2-dp directly correlated with several pro-inflammatory cytokines, confirming that sNOX2-dp was positively associated with an inflammatory environment and negatively associated with immune activation. Circulating cytokines impaired T-cell function, promoting tumor immune escape. Beyond the roles of IL6 and IL1 in creating a favorable microenvironment for tumor growth, the immunosuppressive cytokine IL10, mainly released by Tregs, suppressed lymphocyte activation and increased Treg levels [27]. Additionally, ROS and serum pro-inflammatory cytokines were involved in the cachexia development in lung cancer patients [28], which further reduced immune competence. However, the role of oxygen molecules in activating the immune system remains controversial. In the tumor stroma, ROS produced by cancer-associated fibroblasts (CAFs) promote tumor-supporting activity, partly by recruiting MDSCs [29], which can further limit immune responses by increasing ROS production [30]. ROS, together with the iNOS product NO, formed peroxynitrite, which perturbed T-cell functions by altering the TCR-CD3 complex, decreasing the interaction between TCR and CD8, and rendering T cells unresponsive to antigen-specific stimulation [31]. Similar effects were observed in TAMs or neutrophils, which contributed to lung cancer development [32]. In antigen-presenting cells (APCs) such as DCs, ROS inhibit rapid antigen proteolysis, prolonging its presence in endo/lysosomal compartments and leading to the development of anergic or tolerogenic lymphocytes [33,34]. Conversely, NOX2 played a crucial role in T-cell signaling in autoimmune diseases, enhancing effector functions and Tc1 cytokine production [35]. The different outcomes observed in various settings depended on several key factors: the amount of ROS (with high levels causing DNA and protein damage and low levels triggering cell signaling), the localization of NOX2 (intracellular or extracellular), the duration of ROS release, and, in cancer, the tumor stage that influences the varying requirements for ROS [36]. All these factors affect the oxidative balance, which is relevant for driving the immune response and, concurrently, for cancer progression. Furthermore, the analysis of MDSC and Treg subsets showed a positive correlation between PMN-MDSCs and sNOX2-dp, and a negative relationship between non-suppressive Tregs and sNOX2-dp, further supporting sNOX2-dp’s role in inhibiting the anti-tumor immune response in this group of patients. These findings reflected the trends of these immune populations during therapy. In fact, we observed increasing levels of PMN-MDSCs in non-responder patients with more compromised immune systems and non-suppressive Tregs in the overall population after one cycle of therapy, with pNOX2-dp levels in NR patients rising from a median of 33 pg/mL at T0 to 45 pg/mL at T1. The effects of immunotherapy alone on oxidant status have been minimally studied. Most research has focused on the effects of ROS inducers, such as radio- or photodynamic therapies, when combined with immunotherapy to enhance immune function. In breast cancer, ROS restored cytotoxic activity and reduced immunosuppressive cells; in colorectal cancer, ROS induced T-cell infiltration or M1 polarization. However, all these effects, along with others, were mediated by low oxygen concentration [37]. In this work, we demonstrated that sNOX2-dp levels decreased slightly after the first cycle of immunotherapy, suggesting that anti-checkpoint inhibitors can indirectly influence ROS levels, lower oxidative stress, and thus promote favorable immune modulation in the tumor microenvironment. Additionally, when examining the fold increase in sNOX2-dp from T1 to T0, we observed a significant increase in patients treated with ipi/nivo+CHT compared with the other two therapies. This is likely due to the high inflammation caused by the combination of ipilimumab with nivolumab, which often triggers several toxicities that induce oxidative stress. In contrast, chemotherapy did not appear to affect sNOX2-dp modulation, as patients treated with PEM alone or in combination with chemotherapy showed similar sNOX2-dp levels, as previously reported [38].

Finally, we correlated changes in sNOX2-dp with the response to therapy, and in line with the immune results, we saw an increase in sNOX2-dp only in non-responding patients, which did not correlate with treatment. It is important to note that the therapy response was assessed 3 months after starting treatment, during which patients received two additional cycles beyond the initial one. This indicates that after the immune changes observed at T1, further immune system modulation and changes in sNOX2-dp might have taken place. Despite this limitation, these findings suggest that sNOX2-dp could be a potential predictive marker for response and can be evaluated in metastatic NSCLC patients regardless of treatment.

## 5. Conclusions

This work demonstrated for the first time the trend of sNOX2-dp in metastatic non-oncogene-addicted NSCLC patients undergoing immunotherapy. We found that these molecules contribute to creating an immunosuppressive and inflammatory environment, emphasizing the importance of combining immunotherapy with NOX2/ROS-targeted treatments in these patients. Additionally, monitoring the oxidant status of cancer patients through circulating sNOX2-dp measurements makes this analysis quick and easy, allowing clinicians to track tumor progression during treatment and identify patients more likely to respond.

## Figures and Tables

**Table 1:** Patients’ Characteristics.

	N (%)
	79 (100%)
**Age (median, range)**	67 (48–90)
**Sex**	
Male	51 (65)
Female	28 (35)
**Performance Status (PS)**	
0	42 (53)
1	31 (39)
2	6 (8)
**Smoking status**	
Current smoker	34 (43)
Former smoker	36 (46)
Non-smoker	9 (11)
**Comorbidity**	
yes	50 (64)
no	29 (36)
**Therapy**	
pembrolizumab	31 (39)
pembrolizumab + chemotherapy	27 (34)
ipilimumab + nivolumab+ chemotherapy	21 (27)
**Response to Therapy**	
Yes	52 (66)
No	27 (34)

## Data Availability

The data presented in this study are available on request from the corresponding authors due to privacy.

## Supplementary Materials

The following supporting information can be downloaded at: https://www.mdpi.com/article/10.3390/antiox15030290/s1, Table S1: Correlation between sNOX2-dp at T0 and clinical parameters; Table S2: Correlation between sNOX2-dp and immune cells, both assessed at baseline; Table S3: Correlation between sNOX2-dp T1/T0 and immune cells at T0; Figure S1: Histograms display the mean values + SEM related to the percentage of Ki67CD8, CD137, CD137Ki67, and CD3 T cells in NSCLC patients treated with pembrolizumab (A), pembrolizumab + chemotherapy (B), and ipilimumab + nivolumab + chemotherapy (C). Patients were divided based on the median sNOX2-dp value of 37.61 pg/mL, with groups below (dark grey) and above this threshold (light gray). *p*-values less than 0.05 were considered significant.

## Abbreviations

CPs	cancer patients
ECOG	Eastern Cooperative Oncology Group
HDs	healthy donors
ICIs	Immune Checkpoint Inhibitor
Ipi/Nivo+CHT	ipilimumab + nivolumab + chemotherapy
M-MDSCs	Monocyte-MDSCs
MDSCs	Myeloid-Derived Suppressive Cells
NADPH	Nicotinamide Adenine Dinucleotide Phosphate
NSCLC	non-small-cell lung cancer
PBMCs	Peripheral blood mononuclear cells
PEM	Pembrolizumab
PEM+CHT	Pembrolizumab + Chemotherapy
PMN-MDSCs	Polymorphonuclear MDSCs
PS	performance status
ROS	reactive oxygen species
sNOX2-dp	soluble NOX2-derived peptide
TAMs	Tumor-Associated Macrophages
