# Supplementary material for: Correlation Between Oxidative Stress and Immune Profiles During Immunotherapy in Metastatic Non-Oncogene-Addicted NSCLC Patients
Source: Antioxidants (Basel). 2026 Feb 26;15(3):290. doi: 10.3390/antiox15030290 (PMC13024447; doi:10.3390/antiox15030290)
Supplement: Supplementary file 1 [file antioxidants-15-00290-s001.zip › antioxidants-4148242-supplementary.pdf]

Clinical Parameters	nNox2 (pg/ml) median±SD	P Value
**Age**		0.1
<65	39±17	
≥65	37±12	
**Sex**		0.1
M	37+14	
F	41+14	
**Smoking Status**		0.5
Yes	38+17	
No	37+9	
Ex	38+14	
**Performance Status**		0.7
0	37+15	
1	38+14	
2	39+11	
**Response**		0.5
Yes	38+14	
No	37+15	
**Therapies**		0.4
PEM	37+13	
PEM+CHT	37+14.5	
IPI/NIVO+CHT	38+15	
**ICIs-related toxicity**		0.1
Yes	37+13	
No	39+17	

Supplementary Table S1: Correlation between sNOX2-dp at T0 and clinical parameters

	Immune cell subset	r	95% CI	P value
PEM	CD3	0.43	0.087 to 0.686	0.01
	Effector memory T cells	-0.39	0.05 to 0.67	0.03
PEM+CHT	CD137 T cells	-0.39	-0.67 to -0.008	0.04
	CD137PD1 T cells	-0.41	-0.68 to 0.002	0.03
	CD137 naïve T cells	-0.39	-0.67 to -0.008	0.04
Ipi/Nivo+CHT	CD3	0.5	0.09 to 0.76	0.01
	Ki67 T cells	-0.46	-0.74 to 0.03	0.03
	Ki67CD8 T cells	-0.46	-0.74 to 0.03	0.03
	Ki67CD4 T cells	-0.44	-0.73 to 0.01	0.04
	Ki67 Effector T cells	-0.43	-0.73 to 0.008	0.04

Supplementary Table S2 : Correlation between sNOX2-dp and immune cells, both assessed at baseline.

	Immune cell subset	r	95% CI	P value
PEM	CD137 naïve T cells	-0.5	-0.8 to -0.06	**0.02**
	CD28 naïve T cells	-0.6	-0.8 to -0.2	**0.005**
PEM+CHT	CD137PD1 T cells	-0.6	-0.84 to -0.16	**0.01**
	CD137 naïve T cells	-0.5	-0.83 to -0.05	**0.03**
	PD1 T cells	-0.5	-0.77 to -0.01	**0.04**
	PD1CD8 T cells	-0.5	-0.81 to 0.1	**0.02**
Ipi/Nivo+CHT	CD4 T cells	-0.6	0.86 to -0.29	**0.002**
	Ki67 naïve T cells	-0.5	-0.78 to 0.009	**0.04**

Supplementary Table S3: Correlation between sNOX2-dp T1/T0 and immune cells at T0

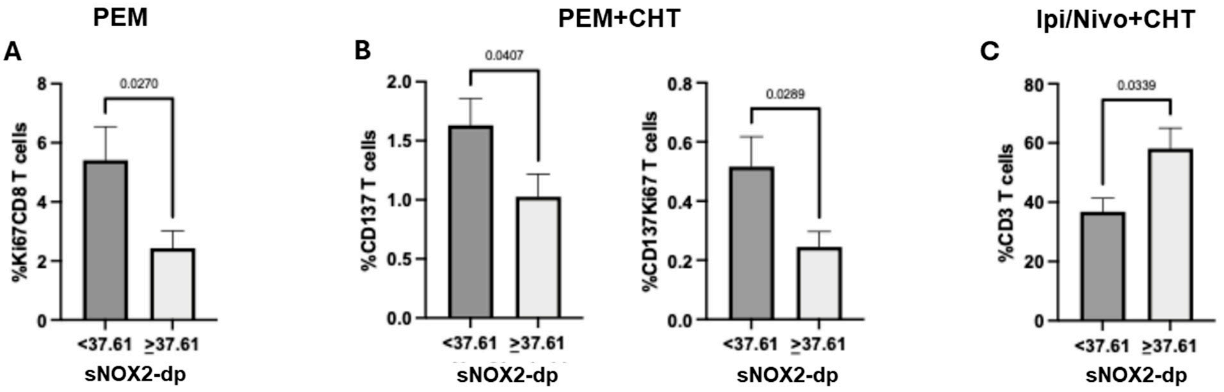

Supplementary Figure S1: Histograms display the mean values ± SEM related to the percentage of Ki67CD8, CD137, CD137Ki67, and CD3 T cells in NSCLC patients treated with pembrolizumab (A), pembrolizumab + chemotherapy (B), and ipilimumab + nivolumab + chemotherapy (C). Patients were divided based on the median sNOX2-dp value of 37.61 pg/mL, with groups below (dark grey) and above this threshold (light gray). P-values less than 0.05 were considered significant.
